# Effects of Gape and Tooth Position on Bite Force and Skull Stress in the Dingo (*Canis lupus dingo*) Using a 3-Dimensional Finite Element Approach

**DOI:** 10.1371/journal.pone.0002200

**Published:** 2008-05-21

**Authors:** Jason Bourke, Stephen Wroe, Karen Moreno, Colin McHenry, Philip Clausen

**Affiliations:** 1 School of Biological Earth and Environmental Sciences, University of New South Wales, Sydney, New South Wales, Australia; 2 School of Environmental and Life Sciences, University of Newcastle, Callaghan, New South Wales, Australia; 3 School of Engineering, University of Newcastle, Callaghan, New South Wales, Australia; University of Wisconsin, United States of America

## Abstract

Models of the mammalian jaw have predicted that bite force is intimately linked to jaw gape and to tooth position. Despite widespread use, few empirical studies have provided evidence to validate these models in non-human mammals and none have considered the influence of gape angle on the distribution of stress. Here using a multi-property finite element (FE) model of *Canis lupus dingo*, we examined the influence of gape angle and bite point on both bite force and cranial stress. Bite force data in relation to jaw gape and along the tooth row, are in broad agreement with previously reported results. However stress data showed that the skull of *C. l. dingo* is mechanically suited to withstand stresses at wide gapes; a result that agreed well with previously held views regarding carnivoran evolution. Stress data, combined with bite force information, suggested that there is an optimal bite angle of between 25° and 35° in *C. l. dingo.* The function of these rather small bite angles remains unclear.

## Introduction

Theoretical models of the mammalian jaw apparatus predict that bite force is intimately linked to jaw gape angle and bite position [1]–[4]. Shallower gapes and more proximal tooth positions should lead to greater overall force production. While some validation has been forwarded with respect to humans,[5] few studies have examined relationships between these factors in other vertebrate species and none have investigated their influence on cranial stress distributions. Many studies have focused only on maximum bite force [6]–[8]. Tests on how force varies along the jaw line, and between gape angles, have been few and far between [9]–[12] and none have concentrated on carnivoran mammals. It has been argued that carnivoran muscle insertion geometry, and mandibular articulation angles might facilitate the generation of greater bite forces at wider angles than in more generalized mammals [12]–[13]. Thus, it is possible that carnivoran skull mechanics don't follow patterns deduced for other taxa.

Finite Element (FE) analysis has become increasingly popular as a means of examining biomechanical questions [13]–[18]. The non-destructive, malleable nature of FE models and their capacity to reveal detailed information has allowed researchers to test biomechanical scenarios that would be extremely time-consuming, dangerous, or ethically challenging using available *in vivo* technologies 19–21.

Our aim in the present study has been to apply a Finite Element approach to examine the affect of both gape angle and bite position on bite force and to map the influence of variation in these factors on cranial stress in a relatively generalized carnivoran mammal. The subject used was an Australian dingo (*Canis lupus dingo*).

## Materials and Methods

We used an FE model of a dingo skull (AM 38587) previously assembled by Wroe et al [22] from computerized serial tomography data. This model comprised eight material properties incorporated on the basis of density data [22]. Seven iterations of this model were generated using Strand7 (Vers. 2.3) in which gape angles differed by 10 degrees, ranging from 65° (maximal gape) to 5°.

Restraint and rigid link assignment were as in Wroe et al [22] To prevent free body rotation, and more broadly distribute force, a framework of rigid links was placed at the occipital condyle, and on tooth bite points. A linear static test was performed on each model. Two bite transmitted load cases were used. They consisted of bites that were driven solely by the skull musculature, with maximal bite force being assumed for each instance. The two intrinsic load cases used were a bilateral bite at the canines, and a bilateral bite at the carnassials. Muscle forces and architecture were approximated through the addition of pretensioned trusses. These are beam elements, that carry axial loads only. Truss element numbers and diameter were as in Wroe et al [22].

Mean brick element stress was obtained from six regions of the skull. These regions were: the entire skull, the cranium (skull sans the mandible), the rostrum (the region from the antorbital fenestra, to the anterior-most tip of the cranium), the anterior orbit (anterior margin of orbit, to anterior fenestra), the zygomatic arch and the mandible. These regions were chosen for the symmetrical stress distributions that they produced under bilateral load cases.

Mechanical behaviour was determined by the visual output of the post-processing software and mean brick stress values. All data was calculated in terms of Von Mises (VM) stress. Von Mises stress is a function of the principal stresses (σ_1_, σ_2_, & σ_3_) that measures how stress distorts material. Failure of ductile material, such as bone, is estimated when VM stress equals the yield strength of the material in uniaxial tension [14]. Since Von Mises stress is proportional to strain energy, [12] it can be used to determine the amount of strain placed on bone under various load cases.

Statistical analysis was performed using a customized program written in RGUI by Karen Moreno.

## Results

### Bite Force

**Figure 1:**
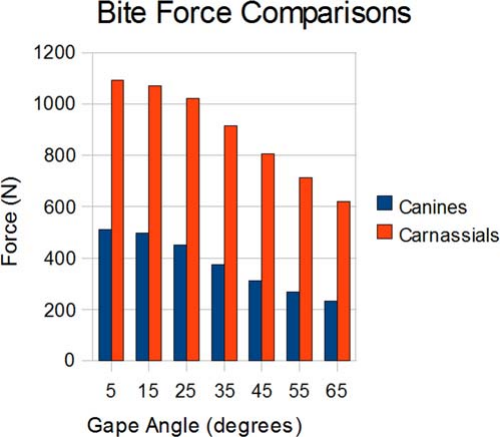
Comparison of canine derived bites, vs carnassial bites.

A negative correlation was found between bite force and gape angle, with shallower angles producing higher bite forces for both canines and carnassials (table 1). Bite force between carnassials and canines varied substantially. On average, carnassial bites produced forces 2.4 times greater than those of canines under similar conditions (fig 1).

**Table 1:** Comparison of gape angle and bite point on overall bite force in C. l. dingo.

Gape Angle	BF Canines	BF Carnassials
65°	231.99 N	620.33 N
55°	269.29 N	712.25 N
45°	312.86 N	806.36 N
35°	374.43 N	916.16 N
25°	450.39 N	1021.43 N
15°	496.79 N	1071.53 N
5°	511.80 N	1091.17 N

### Stress

**Figure 2:**
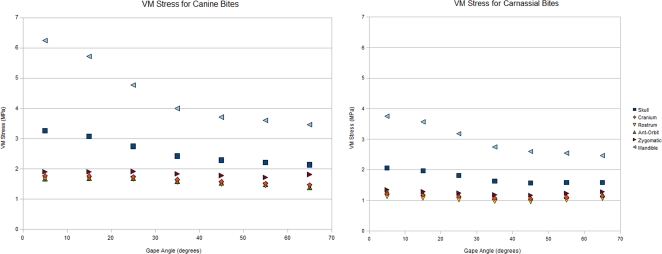
Comparison of stress distribution along the skull during each freedom case.

**Figure 3:**
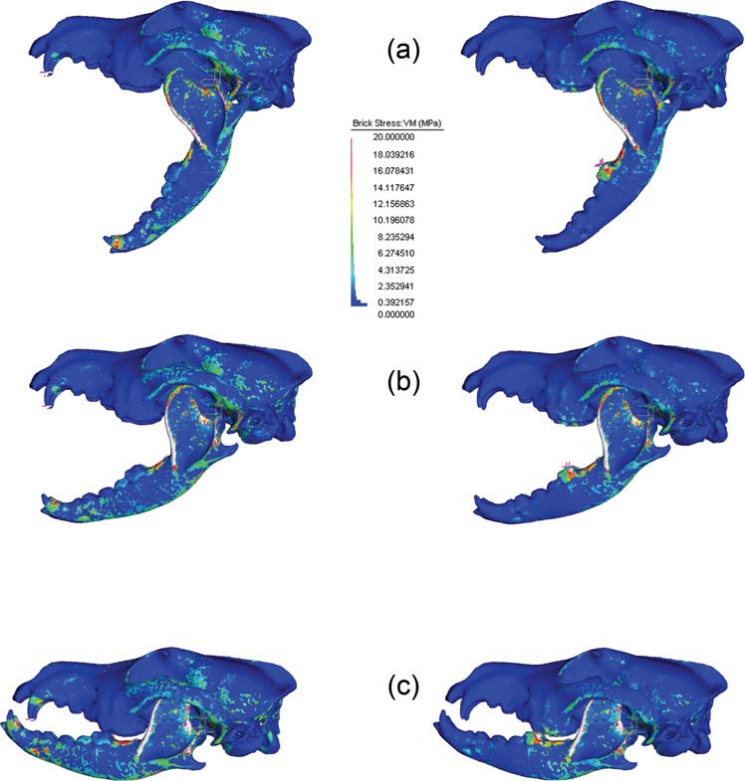
Comparison of stress development in C.l.dingo during bites directed at the canines (left), or carnassials (right) at select angles to show trend. Gape angles are (a&rpar; 65°, (b) 35° and (c) 05°.

Mean brick stress was superficially similar under both load cases. Stress tended to increase as jaw angle decreased (fig 2). The mandible showed the greatest increase in stress out of all the skull regions, with shallower gape angles delivering the largest VM values. VM stress was consistently high along the edges of the coronoid process, and near the base of the mandibular condyle. These stress values were directly muscle related, and could be viewed as an artifact of the muscle modeling [19]. That said, there was a noted increase in stress along these regions of the mandible. This suggested a greater pull being generated by the temporalis and masseter during these shallow bite tests. The zygomatic arch showed the highest VM stress for the cranium (table 2); with stress levels remaining mostly stable throughout both load cases, and all jaw positions. VM stress was always higher in canine bites than respective carnassial bites (fig 3).

**Table 2:** Mean brick stress (VM) of select skull regions during static bite tests at various angles and jaw regions for solved FE models for C. l. dingo.

Gape Angle	Region	Canine	Carnassial
5°	Skull	3.26	2.06
	Cranium	1.77	1.22
	Rostrum	1.69	1.12
	Antorbital	1.68	1.29
	Zygomatic	1.90	1.35
	Mandible	6.24	3.75
15°	Skull	3.08	1.97
	Cranium	1.76	1.17
	Rostrum	1.69	1.07
	Antorbital	1.68	1.22
	Zygomatic	1.91	1.29
	Mandible	5.72	3.57
25°	Skull	2.75	1.81
	Cranium	1.74	1.13
	Rostrum	1.68	1.03
	Antorbital	1.68	1.17
	Zygomatic	1.91	1.24
	Mandible	4.77	3.18
35°	Skull	2.43	1.63
	Cranium	1.64	1.07
	Rostrum	1.58	0.97
	Antorbital	1.60	1.10
	Zygomatic	1.84	1.18
	Mandible	4.00	2.75
45°	Skull	2.29	1.57
	Cranium	1.58	1.06
	Rostrum	1.51	0.96
	Antorbital	1.53	1.08
	Zygomatic	1.78	1.17
	Mandible	3.71	2.60
55°	Skull	2.21	1.58
	Cranium	1.52	1.10
	Rostrum	1.46	1.02
	Antorbital	1.48	1.13
	Zygomatic	1.72	1.23
	Mandible	3.60	2.54
65°	Skull	2.13	1.58
	Cranium	1.47	1.14
	Rostrum	1.42	1.07
	Antorbital	1.38	1.18
	Zygomatic	1.81	1.28
	Mandible	3.46	2.47

**Figure 4:**
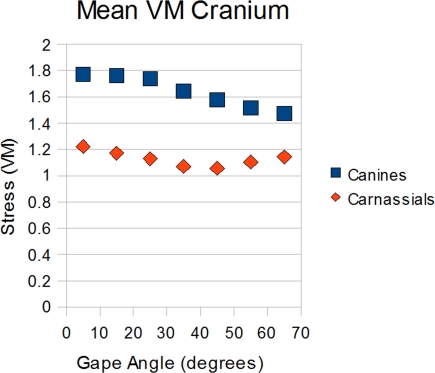
Focus on cranium stress distribution during each freedom case.

Differences between the bite points were also observed in the cranium. For the canine load case, VM stress was seen to increase linearly in the cranium as gape shallowed. However once the jaw reached a 25° angle to the rest of the skull, cranial stress plateaued, with VM stress increasing by no more than 1% between 25° and 5°. The carnassial load case showed a different pattern in the cranium. With the carnassials, there was a notable “dip” in stress starting at 55° and continuing down to 45° where VM values were at their lowest (fig 4).

## Discussion

The data from this study lends support to work using more traditional methods that showed bite force to correlate negatively with jaw gape [4]–[5], [19]. That moving closer to the pivot point of the jaw would increase bite strength is an expected result of the jaw's lever mechanics [11], [23]. Although the largest bite force was generated at the shallowest jaw gape, the greatest *increase* in bite force did not occur at this angle. Rather it occurred at 25° for the canines and 35° for the carnassials.

It is notable that these angles of high bite force increase also appeared to correlate with regions of the cranium that showed either resistance to the increased force (canine bites), or lowered VM stress (carnassial bites). This suggests that the dingo's skull and musculature may be optimized to deliver maximal bites at these angles, with interesting functional implications. Among subspecies of grey wolf and social canids in general, bite force in the dingo is relatively weak and bite force adjusted for body mass allometry has been shown to correlate with prey size [24]–[25]. We suggest that optimal gape angle may also be a useful indicator of feeding ecology among carnivorous mammals. Analyses incorporating both large prey specialists (e.g., *Canis lupus lupus*, *Lycaon pictus)* and small prey specialists (e.g., *Vulpes vulpes*) are needed to examine this proposal. Alternatively it could also be that optimality in this range is necessary for disabling bites, such as the severing of major tendons.

As the mandible is the primary object being powered by the jaw muscles, finding higher VM stress in this skull region is not entirely surprising. It is doubtful, however, that stress increase was solely due to the higher bite force attributed to the more acute angles. If bite force itself was the main driver of mandibular stress, then VM stress in the mandible would be greatest in the much stronger carnassial bites. However, a carnassial bite at maximum gape, producing a force 2.7 times higher than the respective canine bite, resulted in mandibular VM stress that was 30% *lower* than in a canine bite at the same angle. This suggested that dingoes, and perhaps by extension, carnivorans in general, have evolved skulls that are better adapted to tolerate stresses at wider jaw angles than other mammals.

Overall, the results of this study offer support for previous models of mammalian jaw mechanics [1]–[4]. Bite force is markedly affected by both jaw gape and point of contact along the jaw line. Proximal bites and acute jaw angles result in greater overall force. Even for carnivorans, which have undergone evolutionary adaptations to allow for greater bite forces at wider gape angles, the rules of the models remains true.

In conclusion, while bite force in *C. l. dingo* appears to still be limited by overall jaw mechanics, the fact that stress data shows greater tolerance of wide jaw angles, indicates the direction taken in carnivoran evolution. Consideration of stress and bite force data, suggests that there is an optimal bite angle of between 25° and 35° in *C. l. dingo.* Further analyses will be needed to determine whether optimal gape angle might be a useful predictor of feeding ecology.
